# Supplementary material for: Boron-induced transformation of ultrathin Au films into two-dimensional metallic nanostructures
Source: Nat Commun. 2024 Dec 10;15:10518. doi: 10.1038/s41467-024-54464-y (PMC11631948; doi:10.1038/s41467-024-54464-y)
Supplement: Supplementary file 1 — Supplementary Information [file 41467_2024_54464_MOESM1_ESM.pdf]

Supplementary Information

Boron-induced Transformation of Ultrathin Au films into Two-Dimensional Metallic Nanostructures

*Alexei Preobrajenski** [1], *Nikolay Vinogradov* [1], *David A. Duncan* [2], *Tien-Lin Lee* [2], *Mikhail Tsitsvero* [3], *Tetsuya Taketsugu* [3,4], *Andrey Lyalin** [4,5]

[1] *MAX IV Laboratory, Lund University, 221 00 Lund, Sweden*

[2] *Diamond Light Source, Didcot OX11 0QX, UK*

[3] *Institute for Chemical Reaction Design and Discovery (WPI-ICReDD), Hokkaido University, Sapporo 001-0021, Japan*

[4] *Department of Chemistry, Faculty of Science, Hokkaido University, Sapporo 060-0810, Japan*

[5] *Research Center for Energy and Environmental Materials (GREEN), National Institute for Materials Science, Namiki 1-1, Tsukuba 305-0044, Japan*

*e-mail:

Alexei Preobrajenski (Alexei.Preobrajenski@maxiv.lu.se)

Andrey Lyalin (lyalin@icredd.hokudai.ac.jp)

Supplementary Note 1: Growth of Au mono- and bilayers on Ir(111)

Without boron, a single atomic layer of gold grows pseudomorphically on Ir(111), i.e. it adopts the lattice constant of the Ir surface. A lattice-mismatched adsorbate layer would produce a moiré pattern, but it can be seen neither in STM (Supplementary Figure 1) nor in LEED (Supplementary Figure 2b). The strain accumulated in the Au ML is released as a slight corrugation (around 0.15 Å high, see inset in Supplementary Figure 1) forming a pattern of quasi-periodic stripes. A 2 ML thick Au film on Ir(111) is already relaxed towards the bulk Au structure, resulting in the clear moiré patterns both in STM (Supplementary Figure 1b and Figure 2d in the main text) and in LEED (Supplementary Figure 2c).

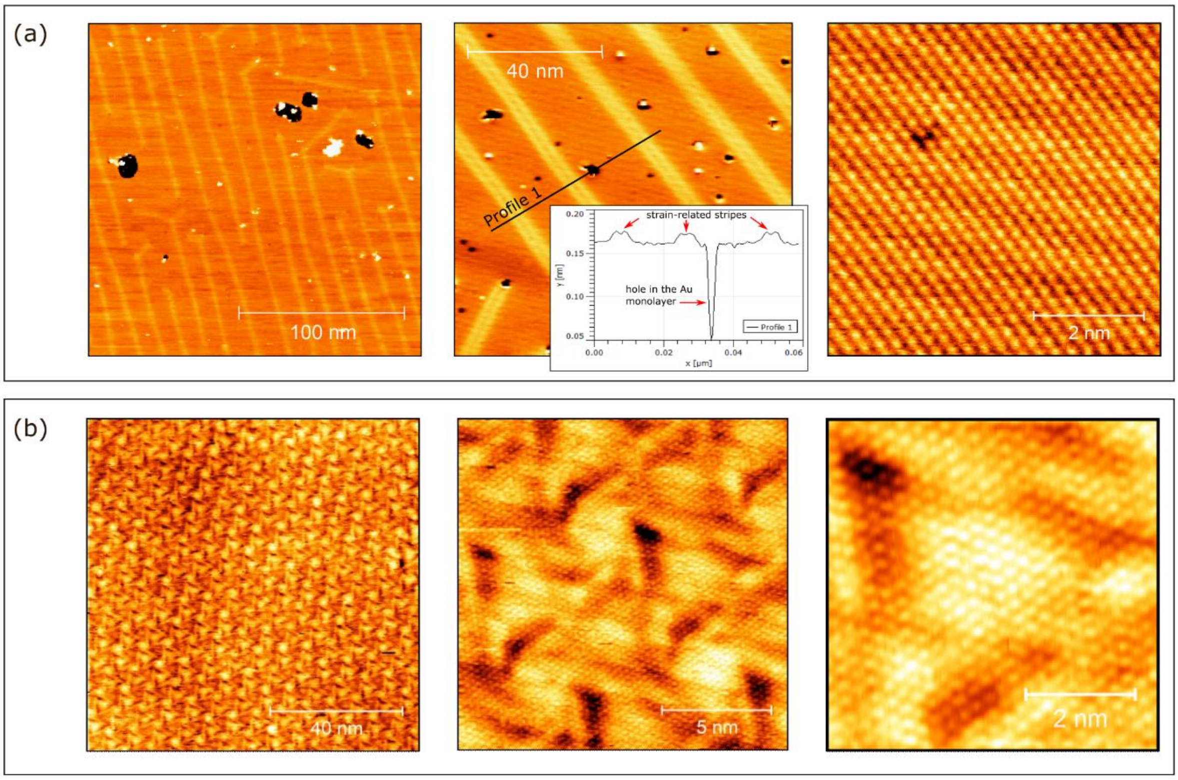

Supplementary Figure 1: STM images of 1 ML (a) and 2 ML (b) of Au grown on Ir(111). *(a): Au lattice is slightly compressed to match Ir lattice. Strain release is visible as slight quasi-periodic elevations, and a height profile across these elevations is show in the inset. No signs of a mismatch-caused moiré pattern can be seen, providing evidence for a pseudo-morphic (strained epitaxial) growth regime. (b): Au lattice is relaxed resulting in a moiré structure with the supercell periodicity of 7.4±0.2 nm.*

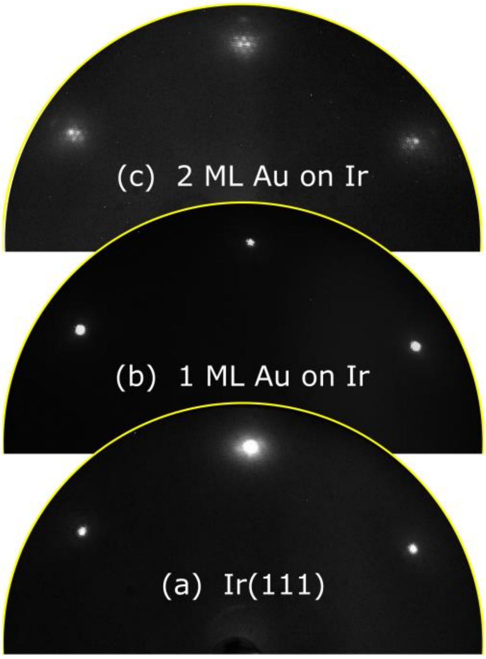

Supplementary Figure 2: LEED patterns *taken with the electron energy of 70 eV from (a) pristine Ir(111) surface, (b) 1 ML Au grown on Ir(111) and (c) 2 ML Au grown on Ir(111). Notice that one ML of gold produces no surface reconstruction confirming the STM observation of a pseudomorphic growth, while two ML thick film results in a clear moiré pattern indicating a partial relaxation of the Au lattice constant towards bulk Au value.*

Supplementary Note 2: STM height profiles for various nanostructured Au films

In Figures 1 and 2 of the main text STM images of 1 and 2 ML Au films on pristine and B-modified Ir(111) surface are presented. For a more direct comparison of the apparent height variation in these structures, we show in Supplementary Figure 3 the measured height profiles through all systems resulting in a moiré structure. It should be noted that we did not observe any significant variations in the image contrast (i.e., in the height profiles) as a function of tunnelling bias voltage and current.

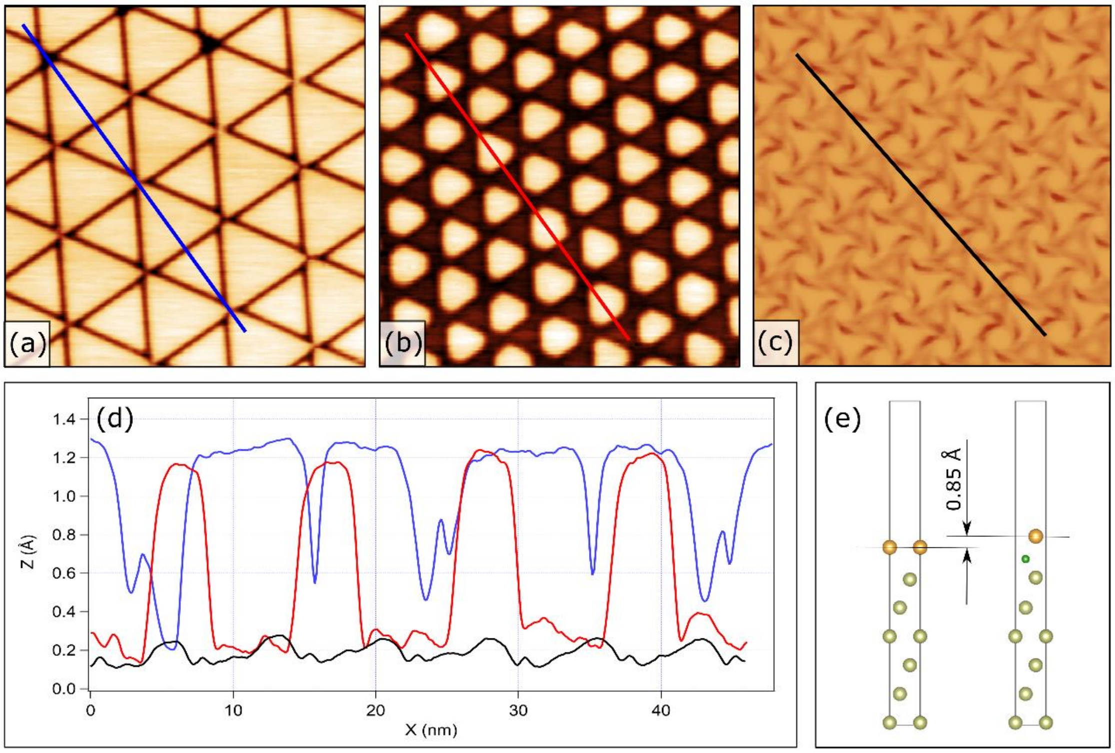

Supplementary Figure 3: Apparent height profiles in various Au/Ir(111) and Au/B/Ir(111) moiré structures. *(a –c) 45 nm large STM images from the (a) 1 ML Au on B/Ir, (b) 2 ML Au on B/Ir and (c) 2 ML Au on Ir. Blue, red and black straight lines denote the trajectory of the height profiles shown in (d), and correspond to the structures (a), (b) and (c), respectively. The colour coding of the profiles in (d) is the same. (e) DFT optimized (1x1) unit cell slabs for 1 ML Au on Ir(111) without and with interfacial boron corresponding to the most energetically favourable atomic arrangements. Atoms are coloured golden (Au), green (B) and grey (Ir). The calculated difference of 0.85 Å in height of the top Au atoms corresponds to the height deviation in (a) and (b).*

The height variations in (a) and (b) are similar, close to 0.9 Å. In (a) it is slightly smaller on average, but this is because the dark tranches are too narrow for the tip to demonstrate full height variation. The variation approaches 0.9 Å in (a) once the profile direction crosses an underdeveloped area at the corner of some triangles. This is very close to the theoretical height difference of 0.85 Å between Au atoms with and without interfacial boron atoms in the HCP positions of Ir(111) (see Supplementary Figure 3e). In the case of a 2 ML Au film grown directly on Ir(111) (c), the height variation is much smaller, below 0.2 Å (compare red and black curves in Supplementary Figure 3d).

Supplementary Note 3: strong sensitivity of borophene on Ir(111) to oxidation

On pristine Ir(111), boron readily adopts a planar configuration, forming 2D sheets known as χ-type borophene [1]. Recent studies have revealed a pronounced sensitivity of these sheets to oxygen, as evidenced by significant changes in the B 1*s* XPS spectra over time [2]. Consistent with these findings, our experiments also demonstrate this susceptibility: the shape of the B 1*s* photoemission line visibly evolves over the course of just one hour even under UHV conditions (refer to Supplementary Figure 4). This oxidation happens due to minute amounts of residual water and O_2 in UHV, resulting in the emergence of a new B 1*s* component (B4) alongside the peaks B1, B2 and B3 attributed to borophene. The high susceptibility to oxidation can be attributed to the catalytic activity of the Ir(111) surface, which facilitates the dissociation of O_2 and H_2O molecules [3], yielding highly reactive atomic O species easily attaching to the borophene sheets. In contrast, buried B films at the Au/Ir interface enjoy enhanced protection due to the presence of a gold overlayer, thereby preserving the shape of the corresponding B 1*s* XPS spectra over time, at least in UHV.

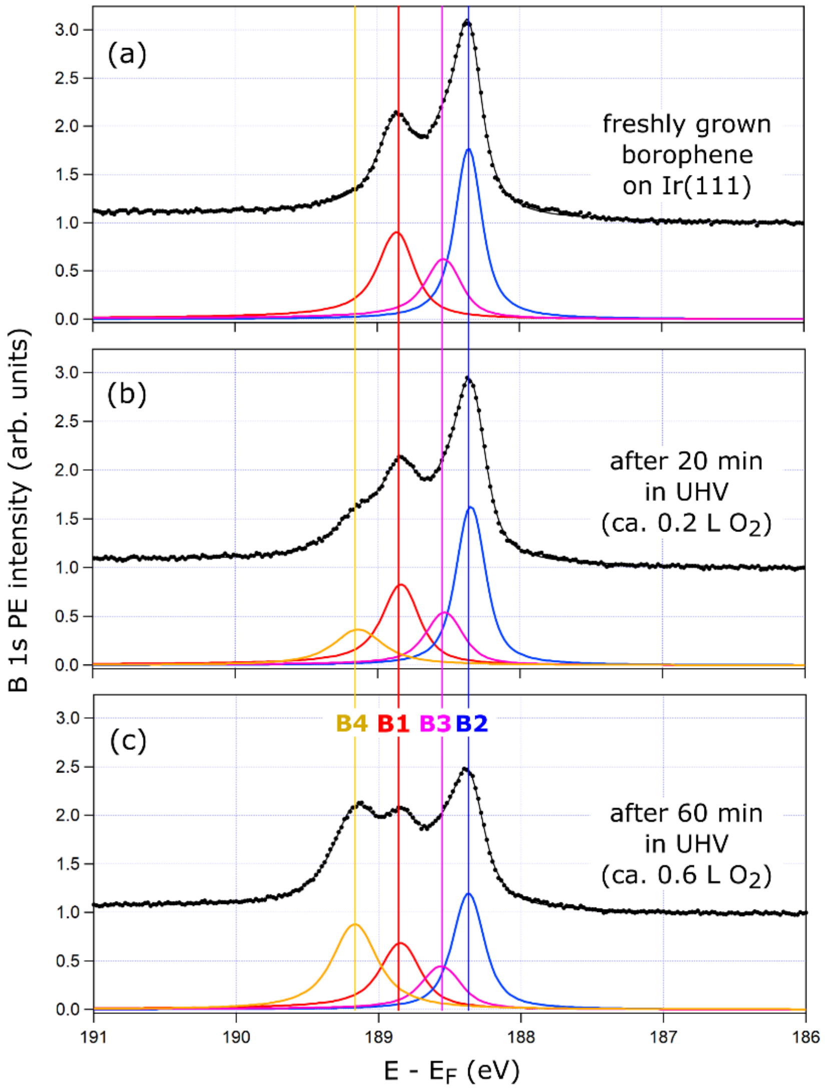

Supplementary Figure 4: Sensitivity of χ-type borophene on Ir(111) to minute amounts of oxygen contaminations *illustrated by the evolution of the B 1s XPS spectrum taken at the photon energy of 270 eV for improved surface sensitivity. (a) pristine borophene right after the growth, (b) after 20 min in UHV (exposed to ca. 0.2 L of residual O_2 and water), (c) after 60 min in UHV (exposed to ca. 0.6 L of residual O_2 and water).*

Supplementary Note 4: Complementary XSW data from buried B layer

To gain insight into the in-plane location of the B atoms in the buried B interlayer, XSW measurements were also performed under grazing incidence geometry, corresponding to the Bragg reflection from the $(1\bar{1}1)$ planes (see Supplementary Figure 5). From the curve fit analysis, the values of the coherent fraction and the coherent position for the B atoms are determined to be $f_{1\bar{1}1}$ = 0.52 ± 0.03 and $p_{1\bar{1}1}$ = 0.68 ± 0.01, respectively. Notably, the value of $f_{1\bar{1}1}$ is substantially smaller than that of f_{111} (0.93 ± 0.04, see main text), suggesting that the B atoms occupy not only a single specific site (HCP, FCC, or atop) but rather two or more distinct sites. DFT calculations (refer to the main text) indicate a minimal energy difference between B atoms occupying the most favourable HCP position and those in the next-most-favourable FCC site. Equal occupation of ideal HCP and FCC hollow sites would yield a $f_{1\bar{1}1}$ of 0.50, and a $p_{1\bar{1}1}$ of 0.76, which compares well with the measured $f_{1\bar{1}1}$ (0.52 ± 0.03) and $p_{1\bar{1}1}$ (0.68 ± 0.01). A 2:1 ratio of hcp:fcc occupancies would result in $f_{1\bar{1}1}$ = 0.58 (or 0.54 if we assume a Debye-Waller factor of 0.93) and $p_{1\bar{1}1}$ = 0.67, which is in even better agreement with the experiment. Therefore, we believe that a mixture of domains with either only HCP or only FCC sites must be present in the grown samples. The co-existence of the HCP and FCC sites within one domain would imply that the honeycomb B layer is formed, but this can be ruled out, because honeycomb B layer would be strongly corrugated and result in a different band structure (see main text for further details).

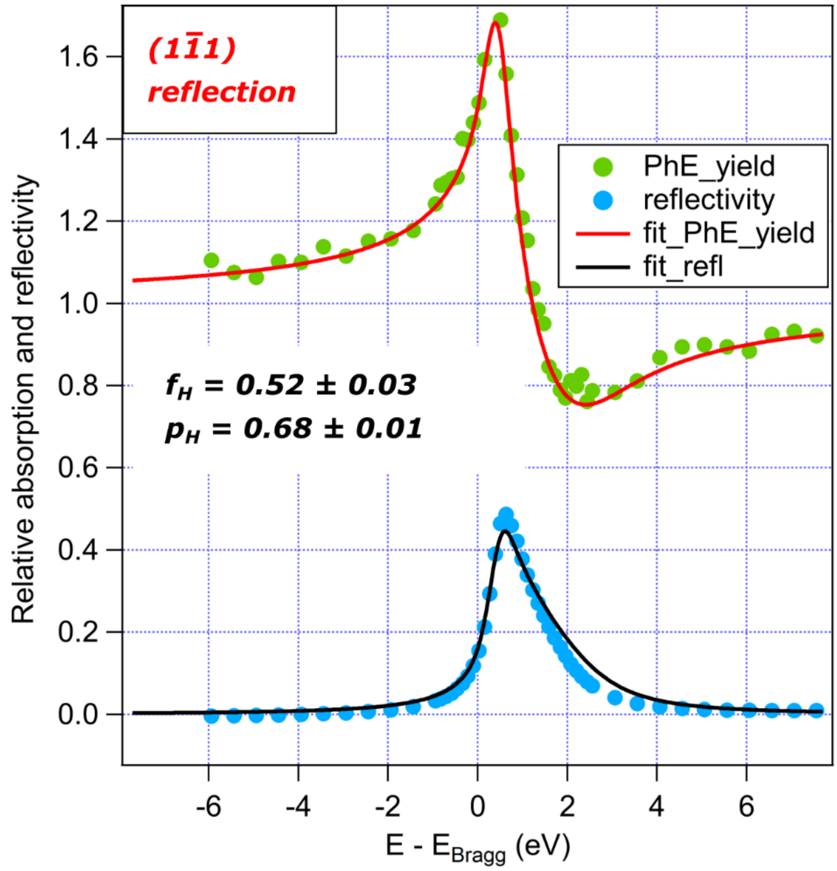

Supplementary Figure 5: Measured and fitted XSW absorption (B 1s photocurrent yield) and reflectivity profiles corresponding to the ***$(1\bar{1}1)$ Bragg reflection*** *from 1 ML Au on B/Ir(111).*

Supplementary Note 5: Optimized geometries from (1x1) unit cell DFT calculations

In Figure 4 (e-g) of the main text the most energetically favourable (1x1)-cell structures are presented for the cases of 1 ML Au on Ir(111), 2 ML Au on Ir(111) and 1 ML Au on B/Ir(111). Below the lattice parameters and atomic coordinates of these structures are listed.

1 ML Au on Ir(111) (Figure 4e of the main text):

```
Lattice parameters (distances in Å, angles in degrees):
   a        b        c       alpha    beta     gamma
 2.71327  2.71327 24.93108  90.0000  90.0000  60.0000

Atomic coordinates (in fractions of the unit cell):
                        x          y          z
   1 Ir   Ir1        0.66667    0.66667    0.44930
   2 Ir   Ir2        0.33333    0.33333    0.36223
   3 Ir   Ir3        0.00000    0.00000    0.27342
   4 Ir   Ir4        0.66667    0.66667    0.18456
   5 Ir   Ir5        0.33333    0.33333    0.09570
   6 Ir   Ir6        0.00000    0.00000    0.00684
   7 Au   Au1        0.00000    0.00000    0.54837
```

2 ML Au on Ir(111) (Figure 4f of the main text):

```
Lattice parameters (distances in Å, angles in degrees):
   a        b        c       alpha    beta     gamma
 2.71327  2.71327 24.93108  90.0000  90.0000  60.0000

Atomic coordinates (in fractions of the unit cell):
                        x          y          z
   1 Ir   Ir1        0.66667    0.66667    0.44940
   2 Ir   Ir2        0.33333    0.33333    0.36252
   3 Ir   Ir3        0.00000    0.00000    0.27342
   4 Ir   Ir4        0.66667    0.66667    0.18456
   5 Ir   Ir5        0.33333    0.33333    0.09570
   6 Ir   Ir6        0.00000    0.00000    0.00684
   7 Au   Au1        0.00000   -0.00000    0.54672
   8 Au   Au2        0.33333    0.33333    0.65206
```

1 ML Au on B/Ir(111) (Figure 4g of the main text):

```
Lattice parameters (distances in Å, angles in degrees):
   a        b        c       alpha    beta     gamma
 2.71327  2.71327 24.93108  90.0000  90.0000  60.0000

Atomic coordinates (in fractions of the unit cell):
                        x          y          z
   1 Ir   Ir1        0.66667    0.66667    0.45520
   2 Ir   Ir2        0.33333    0.33333    0.36175
   3 Ir   Ir3        0.00000    0.00000    0.27342
   4 Ir   Ir4        0.66667    0.66667    0.18456
   5 Ir   Ir5        0.33333    0.33333    0.09570
```

```
6 Ir   Ir6          0.00000     0.00000     0.00684
7 Au   Au1          0.66667     0.66667     0.58237
8 B    B1           0.33333     0.33333     0.51224
```

Supplementary Note 6: Total energies from (1x1) unit cell DFT calculations

In our DFT calculations of 1 ML Au on B/Ir(111), we investigated three distinct arrangements of Au atoms within the Au-B-Ir (1x1) unit cell. In each arrangement, the B atom occupies the HCP site on the Ir surface, while the position of the Au atom above the B layer varies. It can be situated atop the Ir atom of the first layer, above the B atom (e.g. HCP position in respect to the Ir surface), or above the FCC void of the Ir surface. There are also slightly less stable "twins" of these structures, with B sitting in the FCC hole.

The corresponding adsorption energies calculated relatively to the most stable configuration (the Au atom is atop of Ir atom, and the B atom is in HCP position in respect to the Ir surface) are listed below:

Position of Au atom	B (HCP)	B (FCC twin)	B (top)
top	**0.000 eV**	0.059 eV	1.390 eV
HCP	0.119 eV	0.227 eV	1.200 eV
FCC	0.175 eV	0.188 eV	1.195 eV

All listed positions are relative to the first layer of the Ir surface. Here, the most energetically favourable structure was considered the most relevant for comparison with the experiment in Figure 4 in the main text.

Interesting, that in the case of 2ML Au the arrangement of atoms in the most favourable structure is different from that for 1ML Au: B is still sitting in the HCP hole; however, the interfacial Au atom is atop the B atom (HCP position in respect to the Ir surface). The Au atom of the top layer is in the FCC void of Ir surface. The corresponding FCC “twins” with B sitting in the FCC hole are slightly less stable.

Position of the interfacial Au atom	B (HCP)	B (FCC twin)
top	0.056 eV	0.114 eV
HCP	**0.000 eV**	0.070 eV
FCC	0.204 eV	0.267 eV

Supplementary Note 7: Calculated band structures for various B interlayers in the Au/B/Ir heterostructure

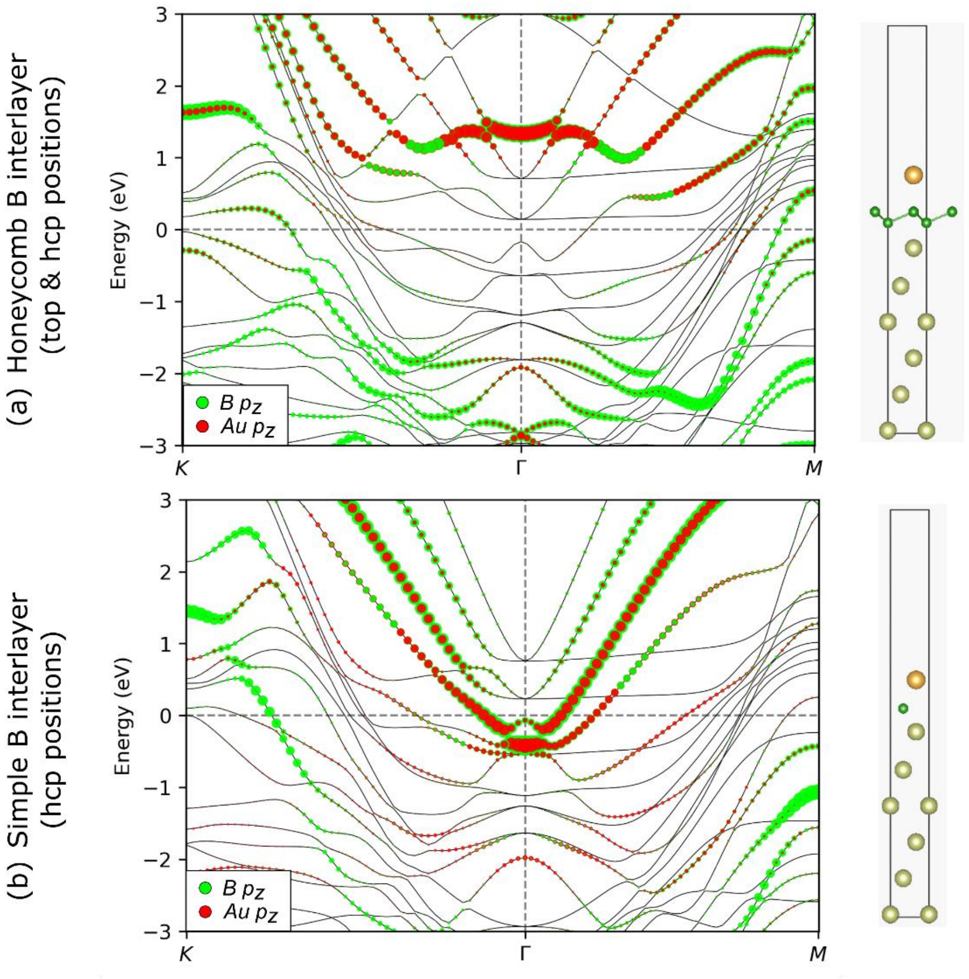

Supplementary Figure 6: DFT calculation of the band structure for the (1x1) unit cell slabs shown to the right. *In each case, there are 6 layers of Ir atoms, one layer of A atoms, and an interlayer of B atoms in-between. In (a) the B interlayer has a honeycomb structure, with one B atom sitting in the atop position and another one – in the HCP hollow site. This structure was found the most energetically stable among various possible honeycomb arrangements. In (b) the interlayer is made of only HCP B atoms; this structure is the most favourable among single-B interlayer structures. Bands highlighted in red and green have predominantly Au p_z and B p_z character, respectively.*

In Supplementary Figure 6, we compare the DFT-calculated band structures for the most stable (1x1) unit cell configurations with two (a) and one (b) B atoms in the boron interlayer. Notably, the band with the mixed Au p_z + B p_z character strongly disperses (nearly parabolic) and intersects the Fermi level only in case (b). The band structure of the honeycomb B interlayer (a) differs significantly from the experimental observation in Figure 4d of the main text, leading to the definitive exclusion of this arrangement. It's worth mentioning that we also explored several less energetically favourable honeycomb configurations (e.g., with the B atoms in the HCP and FCC hollow sites), and the corresponding band structures were similarly distant from the experimental observation.

Atomic coordinates of the honeycomb B interlayer structure calculated in Supplementary Figure 6a:

Lattice parameters (distances in Å, angles in degrees):

a	b	c	alpha	beta	gamma
2.71702	2.71702	24.9652	90.0000	90.0000	60.0000

Atomic coordinates (in fractions of the unit cell):

			x	y	z
1	Ir	Ir1	0.66667	0.66667	0.45463
2	Ir	Ir2	0.33333	0.33333	0.36158
3	Ir	Ir3	0.00000	0.00000	0.27342
4	Ir	Ir4	0.66667	0.66667	0.18456
5	Ir	Ir5	0.33333	0.33333	0.09570
6	Ir	Ir6	0.00000	0.00000	0.00684
7	Au	Au1	0.66667	0.66667	0.63345
8	B	B1	-0.00000	0.00000	0.51642
9	B	B2	0.66667	0.66667	0.54340

Supplementary Note 8: Comparison with the band structure of a freestanding Au monolayer

In Supplementary Figure 7, we show band structures of the Au/B/Ir(111) system calculated for the (1x1) unit cell slab, where B atoms are residing in the HCP hollow sites of the Ir(111) substrate, but Au atoms have different positions. In (a), the Au atom is force-elevated by 10 Å away from the B/Ir substrate in the slab, to represent a freestanding Au monolayer. To enable this elevation, the vacuum layer above the surface has been made thicker resulting in a slab height of 50 Å (instead of 25 Å used normally). In (b), Au is residing in its equilibrium position, as shown in Figure 4g of the main text. For consistency, the same slab of 50 Å in height has been used here too. In both cases we highlight orbital contributions of the highest in energy Au d band (Au 5d_{xz}, violet circles), and the lowest in energy Au p band (Au 6p_z, red circles). Although these calculations are based on the (1x1) unit cell, not considering the full complexity of the observed moiré structure, the overall similarity of the Au-related band structure remains unmistakable. Certain variations in the band structure of these two Au monolayers (such as a disruption of the Au 6p_z band at the K and M points in Supplementary Figure 7b) result from orbital mixing with the B 2p_z states, thus setting a limit to what degree the Au monolayer in the Au/B/Ir system can be considered freestanding. Nevertheless, the comparison in Supplementary Figure 7 reveals that the nanostructured Au monolayer, though not entirely freestanding due to noticeable Au-B interactions, demonstrates properties akin to a two-dimensional metallic monolayer separated from the Ir(111) substrate by the boron layer.

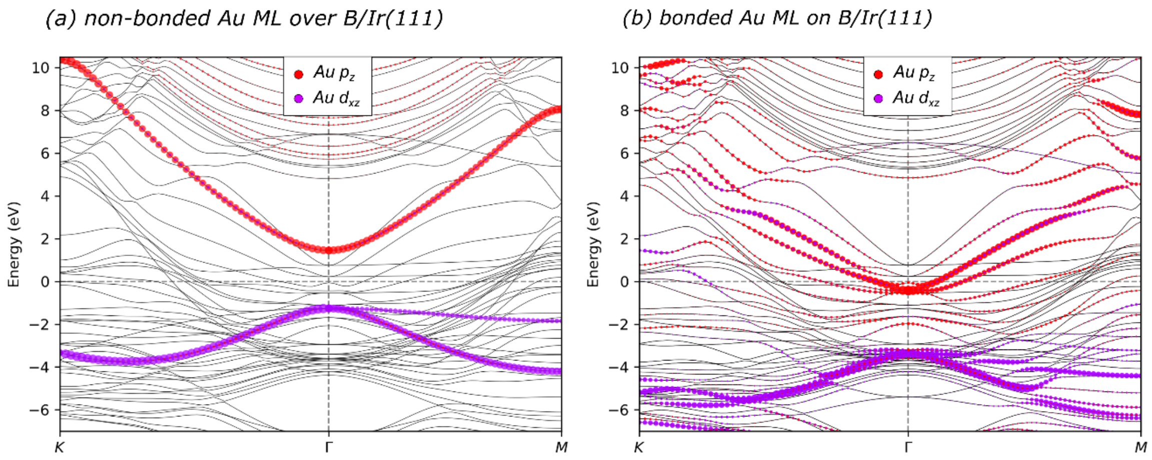

Supplementary Figure 7: DFT band structure of the Au/B/Ir interface calculated for a (1x1) unit cell slab containing a single Au and a single B monolayer. *B atoms are residing in the HCP hollow sites of the Ir(111) substrate. (a) Au atoms are force-elevated from the B/Ir substrate by 10 Å. (b) Au atoms are residing in their equilibrium positions. Red (violet) circles outline the Au 6p_z (Au 5d_{xz}) contribution.*

Supplementary Note 9: Partial density of unoccupied states

The unoccupied fraction of the band structure from Supplementary Figure 6b is shown in Supplementary Figure 8a, with B 2p_{xy} contributions displayed in blue and B 2p_z contributions in red. Supplementary Figure 8b shows the corresponding partial DOS, while Supplementary Figure 8c reproduces experimental differential B 1*s* x-ray absorption spectrum from Figure 3e of the main text. Although the ground state partial DOS alone cannot adequately describe the absorption features quantitatively, it enables us to rationalize why the near-edge absorption structure of the B 1*s* spectrum in the Au/B/Ir heterostructure is predominantly composed of the B 2$p\sigma$* states. This is attributed to the fact that the B 2p_z states reside essentially below the Fermi edge in experiment, while the B 2p_{xy} states constitute the dominating contribution to the unoccupied B-related DOS in the range of several eV above the Fermi level. The exact position of the Fermi level in the DFT calculations in Supplementary Figures 6b and 8a can deviate from the experiment, because the band structures are calculated in the approximation of the (1x1) unit cell, and not the real supercell.

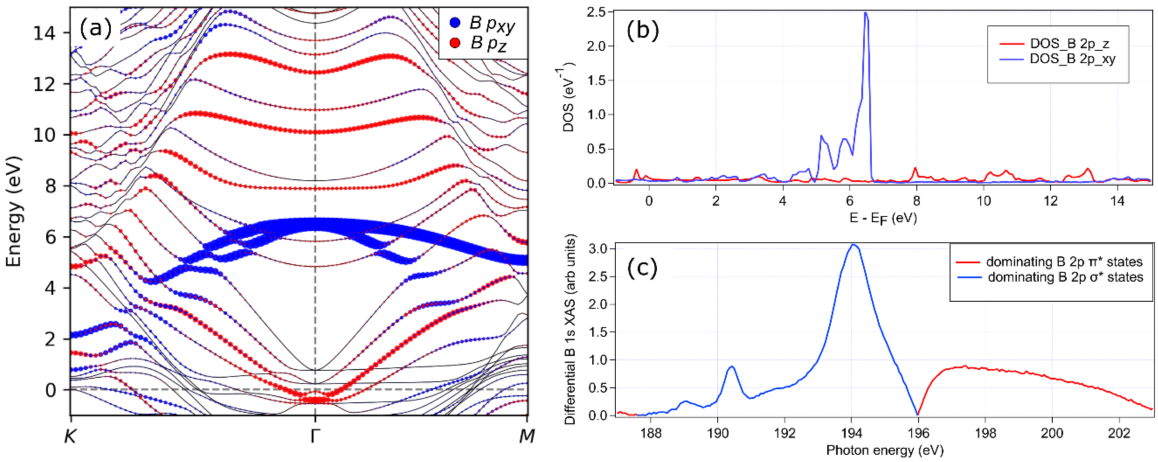

Supplementary Figure 8: Comparison of the calculated ground-state partial density of unoccupied states in the 1 ML Au/B/Ir heterostructure with the measured B 1s NEXAFS. *(a) Band structure of unoccupied states calculated by DFT in the (1x1) unit cell approximation, where red (blue) dots highlight the projection of the out-of-plane B 2*p_z *(in-plane B 2*p_{xy}*) states. (b) corresponding partial DOS. (c) Differential B 1s NEXAFS spectrum (from Figure 3e of the main text).*

Supplementary Note 10: pGFN-FF calculations for Au bilayer on B/Ir(111)

The "dotty" nano-pattern of the 2 ML Au on B/Ir(111) can be rationalized in the context of pGFN-FF calculations. From the STM data, the rhombus supercell parameter of 6.7±0.3 nm corresponds to 24 Ir-Ir distances. Therefore, the following slab was constructed for the calculations: six layers of 24x24 Ir atoms, one layer of 24x24 B atoms in the HCP positions on the Ir substrate and two layers of 23x23 Au atoms. The in-plane displacement of the B atoms caused by the strain accumulated in this slab is shown in the colormap of Supplementary Figure 9a. If we start removing the most "unhappy" (considerably displaced) B atoms from the model, this will result in various kaleidoscopic patterns like the one shown in Supplementary Figure 9b, all different, depending on the threshold used for the B atoms removal. However, regardless of this threshold, there is always one large area per supercell where the remaining B atoms tend to agglomerate. Although the fit with the experimental STM is not perfect, as smaller B clusters do not produce any smaller "dots" in the experiment, the qualitative picture can be reproduced reasonably well. We believe that smaller B islands disappear in the growth process in favour of larger islands because of the kinetically driven ripening.

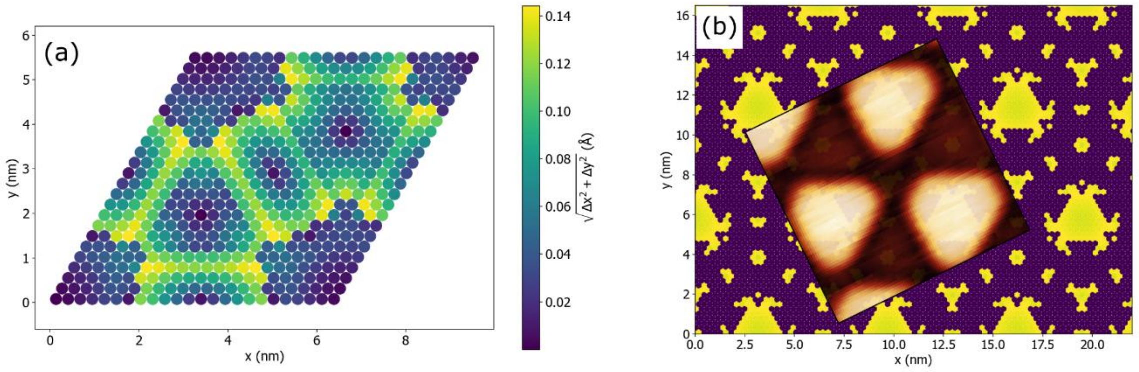

Supplementary Figure 9: Unravelling the nanopattern structure in bilayer gold on B/Ir(111) with pGFN-FF calculations. *Only B atoms are shown. The rhombus supercell was composed of 6 layers of 24x24 Ir atoms, 1 layer of 24x24 B atoms in the HCP positions on the Ir substrate and 2 layers of 23x23 Au atoms. (a): Colormap showing in-plane displacement of individual B atoms from the HCP hollow sites caused by the strain in the Au/B/Ir heterostructure. (b): Structural model of the B interlayer where the considerably displaced B atoms are removed (remaining B atoms are shown in yellow). The threshold of the displacement values used for removing B atoms was adjusted arbitrarily to better reproduce STM data (superimposed).*

Supplementary Note 11: Sensitivity to ambient conditions

The nanostructured gold films (both mono- and bilayers) in the Au/B/Ir heterostructure remain surprisingly stable not only in UHV but also upon exposing them to ambient conditions. As exemplified in Supplementary Figure 10 for the case of 1 ML Au, the nanopatterning in these films remains intact upon exposing samples to air for around one hour. However, the 1 ML thick Au film cannot protect B interlayer from partial oxidation, and UHV annealing of samples exposed to air destroys the periodicity eventually.

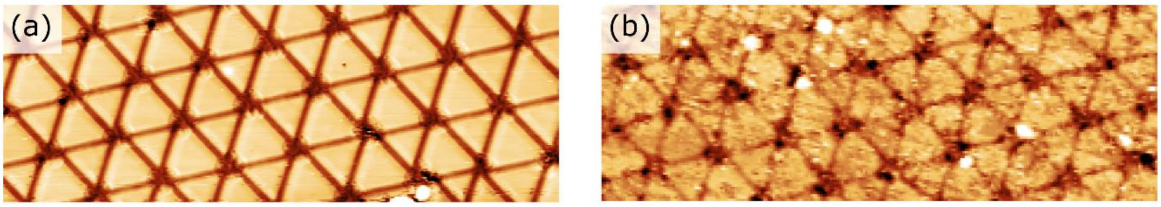

Supplementary Figure 10: exposing nanostructured Au layers to air. *100 x 35 nm STM images from 1ML Au on B/Ir(111) before (a) and after (b) exposing the sample to ambient conditions for approximately 1 hour.*

Supplementary References

[1] Vinogradov, N. A., Lyalin, A., Taketsugu, T., Vinogradov, A. S., Preobrajenski, A., Single-Phase Borophene on Ir(111): Formation, Structure, and Decoupling from the Support. *ACS Nano* **13**, 14511–14518 (2019).
https://doi.org/10.1021/acsnano.9b08296

[2] Kamal, S., Seo, I., Bampoulis, P., Jugovac, M., Brondin, C. A., Menteş T. O., Janković, I. Š., Matetskiy, A. V., Moras, P., Sheverdyaeva, P., Michely, T., Locatelli, A., Gohda, Y., Kralj, M. & Pertović, M., Unidirectional Nano-modulated Binding and Electron Scattering in Epitaxial Borophene. *ACS Appl. Mater. Interfaces* **15**, 57890–57900 (2023).
https://doi.org/10.1021/acsami.3c14884

[3] Bianchi M., Cassese D., Cavallin A., Comin R., Orlando F., Postregna L., Golfetto E., Lizzit S., Baraldi A., Surface core level shifts of clean and oxygen covered Ir(111). *New J. Phys.* **11**, 063002–063020 (2009).
http://doi.org/10.1088/1367-2630/11/6/063002
